# Supplementary material for: Attitude, preparedness, and perceived self-efficacy in controlling COVID-19 pandemics and associated factors among university students during school reopening
Source: PLoS One. 2021 Sep 2;16(9):e0255121. doi: 10.1371/journal.pone.0255121 (PMC8412257; doi:10.1371/journal.pone.0255121)
Supplement: S1 File — (DOCX) [file pone.0255121.s001.docx]

**Information sheet and consent form**

Hello. How are you. My name is_________________. This is an information sheet prepared to conduct a research entitled “Attitude, preparedness, and perceived self-efficacy in controlling COVID-19 pandemics and associated factors among university students during school reopening”. You are randomly selected to participate in this study. You are entirely free to take part or not in this study. The procedure doesn't bear any physical or psychological harm/trauma. Besides, you will not be forced to respond to the information you do not know and want to answer. All information you give will be strictly confidential and kept safe and secure.

Would you like to participate in the study?

1. Yes
2. No

If the participant agrees to participate, thank him/her and provide the questionnaire.

Participant’s Signature__________________ Date____________

Data collector: Name_________________Signature ___________Date____________

If you have any question, you can contact the following persons:

1. Mesfin Tadese Tel: +251 915839921 Email: [mesitad031@gmail.com](mailto:mesitad031@gmail.com)
2. Abebe Mihretie Tel: +251911580160 Email: [abew473@gmail.com](mailto:abew473@gmail.com)

**Questionnaire**

**Part I: Baseline characteristics**

| **S. No** | **Questions** | **Response and coding categories** |
| --- | --- | --- |
| 101 | How old are you? | __________ years. |
| 102 | Sex | 1. Male 2. Female |
| 103 | Where is your original place of residence? | 1. Rural 2. Urban |
| 104 | To which religious group do you belong? | - - - 1. Christian       2. Muslim |
| 105 | What is your current marital status? | 1. Single 2. In a relationship 3. Married 4. Divorced 5. Widowed |
| 106 | What is your faculty? | 1. Health/Medical 2. Non-health/Non-medical |
| 107 | What is your current level of education? | - - - 1. Undergraduate study       2. Postgraduate study |
| 108 | What is your family size? | _________________ |
| 109 | What is the educational level of your mother? | 1. No formal education 2. Elementary school (1-8^th^ grade) 3. Secondary school (9-12^th^ grade) 4. Higher education |
| 110 | What is the educational level of your father? | 1. No formal education 2. Elementary school (1-8^th^ grade) 3. Secondary school (9-12^th^ grade) 4. Higher education |

**Part II:** **Attitude towards COVID-19 prevention measures**

| **Tick your response in the space provided** | | **Agree** | **Neutral** | **Disagree** |
| --- | --- | --- | --- | --- |
| 201 | Wearing a face mask helps to prevent COVID-19. |  |  |  |
| 202 | Avoidance of touching nose, eye, and face with unwashed hand protect COVID-19. |  |  |  |
| 203 | Do you think avoiding handshake and kissing with others protect COVID-19? |  |  |  |
| 204 | Physical distancing helps to prevent COVID-19. |  |  |  |
| 205 | Do you think frequent hands washing with soap and water prevents COVID-19? |  |  |  |
| 206 | The use of hand sanitizers helps to prevent COVID-19. |  |  |  |
| 207 | Keeping rooms well ventilated helps to prevent COVID-19. |  |  |  |
| 208 | Avoiding crowds helps to prevent COVID-19. |  |  |  |
| 209 | Coughing into a bent elbow or tissue helps prevent COVID-19. |  |  |  |
| 210 | Self-quarantine of suspected peoples helps to prevent COVID-19. |  |  |  |

**Part III: Preparedness to combat the spread of COVID-19**

| **Tick your response in the space provided** | | **Yes** | **No** |
| --- | --- | --- | --- |
| 301 | Wearing a face mask at all times in public spaces. |  |  |
| 302 | Readiness to avoid touching eye, nose, and face with an unwashed hand. |  |  |
| 303 | Avoid handshakes and kiss with others. |  |  |
| 304 | Maintain physical distance |  |  |
| 305 | I am frequently cleaning hands with soap and water. |  |  |
| 306 | Readiness to use hand sanitizers. |  |  |
| 307 | Keeping rooms well ventilated. |  |  |
| 308 | Readiness to avoid crowds. |  |  |
| 309 | Cough into a bent elbow or tissue. |  |  |
| 310 | Readiness to self-quarantine if exposed |  |  |

**Part IV:** **Perceived self-efficacy in controlling COVID-19**

| **Tick your response in the space provided** | | **Agree** | **Neutral** | **Disagree** |
| --- | --- | --- | --- | --- |
| 401 | I believe I can protect myself against COVID-19. |  |  |  |
| 402 | I believe COVID-19 can finally be  successfully controlled. |  |  |  |
| 403 | I can strictly follow prevention behaviors. |  |  |  |
| 404 | I have confidence that Ethiopia can win the battle against COVID-19. |  |  |  |

**Part V: Chronic diseases and history of COVID-19 infection**

| **S. No** | **Questions** | **Responses and coding** |
| --- | --- | --- |
| 501 | Do you have a pre-existing chronic illness? (You can select more than options). | 1. Not at all 2. Diabetes 3. Hypertension 4. Kidney disease 5. Heart disease 6. Respiratory disease 7. HIV 8. Other specify___________ |
| 502 | Do you have a history of coronavirus infection? | 1. Yes 2. No |
| 503 | Do you have friends/family who has tested positive for COVID-19? | 1. Yes 2. No |
| 504 | Do you have friends/family who died from the virus? | 1. Yes 2. No |

**Thank you for co-operation!**
