# Supplementary material for: Attitude, preparedness, and perceived self-efficacy in controlling COVID-19 pandemics and associated factors among university students during school reopening
Source: PLoS One. 2021 Sep 2;16(9):e0255121. doi: 10.1371/journal.pone.0255121 (PMC8412257; doi:10.1371/journal.pone.0255121)
Supplement: S2 File — (PDF) [file pone.0255121.s002.pdf]

ደብረ ብርሃን ዩኒቨርሲቲ
Debre Berhan University

"From the community to the community"

ACADEMIC RULES AND REGULATIONS OF DEBRE BERHAN UNIVERSITY FOR CRASH PROGRAM DURING COVID-19

OCTOBER 2020

DEBRE BERHAN

ETHIOPIA

Preamble

Recognizing the rapid transmission of Covid-19 pandemic and the multifaceted challenges it is posing to the human race;

Cognizant of the need to mitigate its transmission, minimize risk to the university community and beyond;

Following government's decision to re-open universities with appropriate risk mitigation measures in place;

Understanding the necessity of adopting a university guideline that are consistent with the different directives and protocols issued by authorities at national level on matters related with the re-opening of universities during Covid-19 pandemic;

Whereas, the university found it necessary to revise parts of the University legislation that deals with academic rules and regulations in light of the new context that arises with the outbreak of Covid-19 pandemic;

Now, therefore, the Senate of Debre Berhan University, in accordance with the power vested in it under Article 49(3) of the Higher Education Proclamation No.1152/2019, article 6(1(c)) of Regulation No. 210/2011 and Regulation No. 228/2011, hereby issues this academic rules and regulations of Debre Berhan University for crash program during Covid-19.

Article 1: Short title

This academic rules and regulation may be cited as 'Academic Rules and Regulations of Debre Berhan University for crash program during Covid-19'.

Article 2: Definition

In this guideline, unless the context requires otherwise:

1. "University" shall mean Debre Berhan University.
2. "Student" shall mean any person admitted and registered at the University in the regular, Continuing Education Program (CEP) or any other program.
3. "Academic Rules and Regulations" shall mean the Academic Rules and Regulations of Debre Berhan University during Covid-19'.
4. "Covid-19" shall mean is an infectious disease caused by a newly discovered coronavirus.
5. "Crash program" shall mean an intensive semester of six weeks, save for health and medicine programs, designed to complete the second semester of 2019/20 academic year that was interrupted by Covid-19 closure.
6. "Ministry" shall mean the Ministry of Science and Higher Education Institutions.

7. “Directive” shall mean the directive issued by the Ministry in 2020 for Higher Education during Covid-19.
8. “Legislation” shall mean Debre Berhan University Senate Legislation, 2012.
9. “Senate” shall mean the senate of Debre Berhan University.
10. “Proclamation” shall mean the Higher Education Proclamation No.1152/2019.
11. “Academic Calendar” shall mean the time allotted to the teaching-learning process as approved by the Senate.
12. “Staff” shall mean the academic, administrative and technical staff of Debre Berhan University.
13. Unless the context provides otherwise in this directive, provisions stated in the masculine shall also apply to the feminine.

Article 3: Issuing Authority

This Directive is issued by the Senate of Debre Berhan University pursuant to the powers vested in it by Article 49(3) of the Higher Education Proclamation No.1152/2019, article 6(1(c)) of Regulation No. 210/2011 and Regulation No. 228/2011.

Article 4: Scope

Unless expressly specified otherwise in this academic rules and regulations, the provisions of academic rules and regulations shall apply to the staff and undergraduate students of the university during Covid-19 pandemic.

Article 5: Academic Calendar

1. The length of academic calendar for the semester shall be six weeks; four weeks of classes and two weeks of exam.
2. Notwithstanding with sub-article 1 of this article, the length of calendar for health and medicine programs shall be eight weeks.
3. An academic unit may request the Academic Vice President for the extension of the academic calendar provided under exceptional circumstances, which shall have effect upon the approval of the Senate.

Article 6: General measures

With the aim to mitigate Covid-19 transmission while continuing the teaching-learning process, the following measures shall be strictly adhered by the staff and the students:

1. In-person classroom instruction shall not exceed 30 persons. This shall also apply to laboratories, workshops, tutorials or any other instructional settings.
2. Notwithstanding to sub-article 1 of this article, the class size may exceed 30 persons for large class rooms with strict observance to the social distancing rules in this academic rules and regulations.
3. All students and staff should maintain social distancing in classroom, library, laboratory, workshops or other instructional settings; meaning 1 to 1.5 meters of physical distance apart while social distancing between the instructor and the students in classroom shall be 1.5 to 2 meters.
4. Acceptable face coverings for Covid-19 that are fit for purpose, such as surgical masks, homemade sewn, and face shields covering both the mouth and nose, be worn at all times in public spaces, both in classrooms and outside classrooms.
5. All students and staff are required to wash hands with soap and running water for at least 20 seconds or use hand sanitizer with 70%+ alcohol when entering and leaving class rooms, laboratories, workshops or any other shared spaces.
6. Staff and students with symptoms associated with Covid-19 shall stay in their place of residence and report or seek medical attention.
7. Classrooms shall be ventilated for at least 15 minutes after the end of each session. Classrooms shall be cleaned and disinfected at least twice a day and a minimum of one hour ventilation after disinfection.
8. Responsible bodies shall clean and ventilate offices, classrooms, libraries and laboratories and workshops regularly for at least an hour a day.
9. Group activities such as team-based sports or games are prohibited.
10. Every staff shall devote his full working time to the institution and commit themselves to support students.
11. Measures specified under this article shall also apply to the post-graduate program.

Article 7: Course delivery

1. Flexible instructional modalities that blend in-person and online or virtual learning shall be encouraged.
2. The depth and breadth of course coverage shall take, among other things, the duration of the academic calendar and shall focus on key learning competencies.
3. In order to reduce the risk of exposure, in-person classes shall be limited to 40 minutes session. There shall be at least 15 minutes ventilation before the next session kicks off.
4. Course delivery rules provided under this article shall also apply to the post-graduate program.

Article 8: Assessment and evaluation

1. Assessment and evaluation and its modalities shall be informed by the unique circumstances that the outbreak of the Covid-19 pandemic entails.
2. The content of the assessment or evaluation shall focus on key learning competencies of the course.
3. Paper-based assessment and evaluation shall be minimized to the extent possible, whereas digital assessment or evaluation is strongly encouraged.
4. Unless the nature of the course demands otherwise, Continuous assessment and final exam shall carry equal weight of 50 percent each, which are used to determine the grade points of a course. However, 30 percent of the continuous assessment shall be collected before their return to University in regards to the crush program.
5. Senior essay or project works shall be conducted in a manner that does not expose them to Covid-19. Further guideline may be issued by the senate.
6. Re-examination shall be administered within a week after the end of the final examination.

Article 9: Disciplinary breach

1. Failure to observe the mandatory provisions of this academic rules and regulations shall constitute as breach of discipline.
2. Disciplinary measures shall be taken in accordance with the directive No.31/2020 issued by the Ministry, the legislation of the University or any other relevant laws.

Article 10: Miscellaneous provision

1. This academic rules and regulations shall be read in conjunction with directives No.31/2020 issued by the Ministry or other relevant authorities on matters regarding the reopening of universities during Covid-19 pandemic and general health guidelines issued by the government.
2. No rules or practices of the university shall, in so far as they are inconsistent with the provisions of this academic rules and regulations, have effect with respect to matters covered by this academic rules and regulations.
3. The University legislation shall continue to govern matters that are not covered by this academic rules and regulations.

Article 11: Effective date

1. This academic rules and regulations shall come in to force as of as of 7th October 2020.
2. This academic rules and regulations shall be effective for the duration of the crash program.
