# Supplementary material for: Attitude, preparedness, and perceived self-efficacy in controlling COVID-19 pandemics and associated factors among university students during school reopening
Source: PLoS One. 2021 Sep 2;16(9):e0255121. doi: 10.1371/journal.pone.0255121 (PMC8412257; doi:10.1371/journal.pone.0255121)
Supplement: Attachment — Submitted filename: Remarks .docx [file pone.0255121.s004.docx]

## Firsltly let me thank the authors for the great work they have provided, the work is excellent and is has been an enjoyable experiencing reading it. The topic is of high interest which they have brought, however they may consider looking unto these few remarks. All the best.

## Introduction

The authors may consider talking about second wave of Covid-19. If the authors may provide an evidence of the rules laid down by the university as citation of the memo or any document where these rules are laid will add value to the paper.

## Methodology

What was the criteria for scoring like 80%above is better/good, the authors may decide to provide justification?

## Data analysis

Well written

## Results

The authors should consider rearranging their results presentation to suite with the topic; (Attitude, preparedness, and perceived self-efficacy in controlling COVID-19 pandemics

and associated factors among university students during school reopening) for consistency sake, they should start with attitude then ……

Table 1, kindly they should indicate the level of p-values by putting star(*).

Table 6,7 and 8: below the table, the authors may consider indicating that….. NB: Bold figures represent/mean………

## Discussion

Well written

## Conclusion

Well written

## Limitation

The authors may also consider highlighting a limitation of their findings as they only based on one University in Ethiopia.

## References

Well Updated
